# Immunization of cattle with a *Rhipicephalus microplus* chitinase peptide containing predicted B-cell epitopes reduces tick biological fitness

**DOI:** 10.1017/S0031182024000143

**Published:** 2024-08

**Authors:** María Martina Esperanza Perez-Soria, Daniel Gustavo López-Díaz, Rafael Jiménez-Ocampo, Gabriela Aguilar-Tipacamú, Massaro W. Ueti, Juan Mosqueda

**Affiliations:** 1 Immunology and Vaccines Laboratory, College of Natural Sciences, Autonomous University of Queretaro, Queretaro, QT, Mexico; 2 Master's Program in Sustainable Animal Health and Production, College of Natural Sciences, Autonomous University of Queretaro, QT, Mexico; 3 Campo Experimental Valle del Guadiana, INIFAP, Durango, DG, Mexico; 4 CA Salud Animal y Microbiologia Ambiental, College of Natural Sciences, Autonomous University of Queretaro, QT, Mexico; 5 Animal Diseases Research Unit, Agricultural Research Service, US Department of Agriculture, Pullman, Washington, 99164, USA

**Keywords:** B-cell epitope, chitinase, immunization, *Rhipicephalus microplus*, tick vaccines

## Abstract

*Rhipicephalus microplus*, the cattle fever tick, is the most important ectoparasite impacting the livestock industry worldwide. Overreliance on chemical treatments for tick control has led to the emergence of acaricide-resistant ticks and environmental contamination. An immunological strategy based on vaccines offers an alternative approach to tick control. To develop novel tick vaccines, it is crucial to identify and evaluate antigens capable of generating protection in cattle. Chitinases are enzymes that degrade older chitin at the time of moulting, therefore allowing interstadial metamorphosis. In this study, 1 *R. microplus* chitinase was identified and its capacity to reduce fitness in ticks fed on immunized cattle was evaluated. First, the predicted amino acid sequence was determined in 4 isolates and their similarity was analysed by bioinformatics. Four peptides containing predicted B-cell epitopes were designed. The immunogenicity of each peptide was assessed by inoculating 2 cattle, 4 times at 21 days intervals, and the antibody response was verified by indirect ELISA. A challenge experiment was conducted with those peptides that were immunogenic. The *chitinase* gene was successfully amplified and sequenced, enabling comparison with reference strains. Notably, a 99.32% identity and 99.84% similarity were ascertained among the sequences. Furthermore, native protein recognition was demonstrated through western blot assays. Chitinase peptide 3 reduced the weight and oviposition of engorged ticks, as well as larvae viability, exhibiting a 71% efficacy. Therefore, chitinase 3 emerges as a viable vaccine candidate, holding promise for its integration into a multiantigenic vaccine against *R. microplus.*

## Graphical abstract

**Figure:**
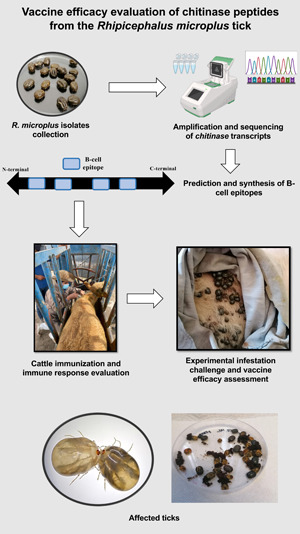

## Introduction

Ticks are haematophagous parasites that harm both humans and animals. These arthropods give rise to significant health issues and substantial economic losses (Anderson and Magnarelli, 2008; de la Fuente *et al*., 2008). The primary tick species affecting cattle is *Rhipicephalus microplus*, which is distributed throughout most tropical and subtropical regions worldwide (Barker and Walker, 2014; Sonenshine and Roe, 2014; Tan *et al*., 2021). In the Americas, *R. microplus* can be found in tropical and subtropical areas of most of the countries, and this area is gradually increasing because of climate change and cattle mobilization (Estrada-Peña *et al*., 2022). The most critical health concern associated with this tick is its role as a vector for pathogenic microorganisms, such as *Babesia bovis*, *B. bigemina* and *Anaplasma marginale*, leading to reduced animal production, severe illness and even death (Bock *et al*., 2004; Almazan *et al*., 2018; Ferreira *et al*., 2022). The economic impact of *R. microplus* on the Mexican livestock industry is estimated at yearly losses of 573.6 million dollars, attributed solely to the tick's direct effect on cattle. However, this appraisal does not account for the costs of tick-borne diseases or the invaluable factor of hindering genetic quality improvement (Rodríguez-Vivas *et al*., 2017).

The control of *R. microplus* remains an urgent issue that still needs to be addressed. For decades, acaricides have been the main tick control strategy. Nevertheless, the emergence of acaricide-resistant tick strains, along with high economic costs and chemical residue pollution, are significant drawbacks to this approach (Rodríguez-Vivas *et al*., 2018; Tiffin *et al*., 2022). Consequently, the need to replace or complement this control method is paramount (Graf *et al*., 2004; Kiss *et al*., 2012). An encouraging alternative is the immunological control based on vaccines. Bm86 recombinant anti-tick vaccines have shown favourable outcomes in various countries, progressively reducing tick populations by disrupting the parasite life cycle and altering reproductive parameters, while also being better in cost-effectiveness (de la Fuente *et al*., 1998; Redondo *et al*., 1999; de la Fuente *et al*., 2007). However, the heterogeneity of Bm86 from different *R. microplus* strains has been reported as a major factor for reduced vaccine efficacy. Molecular and field studies determined that gene variations ranging 3.4–8.6% were sufficient to decrease vaccine efficacy up to 10%, showing an inverse correlation between antigen variation and vaccine efficacy (García-García *et al*., 1999; Bishop *et al*., 2023). The search for antigens that could integrate new tick vaccines has led to the discovery and evaluation of physiologically essential proteins, but only a few have demonstrated effectiveness and undergone field testing (Guerrero *et al*., 2012; de la Fuente and Contreras, 2015; Abbas *et al*., 2023). A potential solution is the development of a new-generation vaccine focused solely on immunological determinants of the antigens, such as B- and T-cell epitopes, rather than employing complete recombinant proteins (Patarroyo *et al*., 2002; Rodriguez-Mallon *et al*., 2015; Scoles *et al*., 2022).

Chitinases constitute a family of enzymes key for the development of arthropods and related to the apolysis and ecdysis phases. These enzymes play a crucial role in the successful moulting between larval to nymphal and nymphal to adult stages, by hydrolysing the chitin polymers of the exoskeleton (Kimura, 1973; Arakane and Muthukrishnan, 2010). One chitinase from *Haemaphysalis longicornis* has been evaluated as a recombinant vaccine antigen, yielding encouraging results. Chitinase vaccination resulted in a 20% reduction in the moulting rate between nymphal and adult stages, an increase in the duration of moulting periods, and a reduction in the tick individual oviposition (You and Fujisaki, 2009).

Our main objective was to identify peptides with predicted B-cell epitopes, in a chitinase from *R. microplus*. Subsequently, after determining the immunogenicity of each peptide, a challenge was performed in immunized and control cattle to determine their ability to reduce the biological fitness of ticks.

## Materials and methods

### Chitinase sequence selection

The chitinase amino acid sequence utilized in this study was chosen through a rigorous search process in the UniProt database (https://www.uniprot.org/) and the National Centre for Biotechnology Information database (NCBI) (https://www.ncbi.nlm.nih.gov/).

### Tick strains

Two distinct *R. microplus* strains were used in this study. The first one, the Queretaro strain, was sourced from different farms in Queretaro state, Mexico. The second one is the Media Joya strain, maintained under laboratory conditions. A minimum of 30 adult female ticks from each strain were carefully collected alive, disinfected and frozen at −70°C to preserve their integrity.

### Nucleic acids extraction and cDNA synthesis

For DNA extraction, complete ticks were ground in liquid nitrogen and processed with the DNeasy Blood & Tissue Kit (Qiagen, Hilden, Germany), according to the manufacturer's instructions. RNA was obtained using the EZ-10 Spin Column Total RNA Mini-Prep Kit (BioBasic, Markham, ON, Canada). The cDNA was synthesized from extracted mRNA using the SuperScript III First-Strand Synthesis System (RT)-PCR Kit (Invitrogen, Carlsbad, CA, USA) following the manufacturer's guidelines.

### Amplification and sequencing of chitinase gene

The *chitinase* gene amplification was accomplished through an endpoint PCR reaction. The following primers were employed for amplification: 5’-CAGCTAACGCTACCACAGC-3’ (sense) and 5’-TACGGGCACTTTCCTTCGTC-3’ (antisense). The reference sequence was ‘transcribed RNA’, with accession number GBBR01000100.1. The 20 *μ*L PCR reaction mixture contained the following ingredients: 1 *μ*L of cDNA, 1 *μ*L of 10 *μ*M of each primer, 10 *μ*L of PCR Master Mix (Promega, Madison, WI, USA) and 7 *μ*L of nuclease-free water. To ensure the integrity of the experimental setup, DNA samples from each strain with VDAC primers served as the positive control, while water was used as the negative control. PCR was performed under the next amplification conditions: initial denaturation at 95°C for 3 min, followed by 34 cycles of denaturation at 57°C for 30 s, alignment at 95°C for 30 s and extension at 72°C for 1 min. A final extension step was set at 72°C for 5 min. The resulting 975 bp amplicons were visualized using 1.5% agarose gel electrophoresis. The amplicon bands were cut from the agarose gel and were purified with the Wizard® SV Gel and PCR Clean-Up System kit (Promega). The purified DNA was then dispatched to the Institute of Biotechnology (IBT) of the Autonomous University of Mexico for Sanger sequencing. Later, 890 bp were obtained and assembled with the Chromas 2.6.5 software (www.technelysium.com.au).

### Bioinformatics analysis

#### Consensus sequences and multiple alignment

An open reading frame (ORF) search was conducted on the assembled nucleotide sequences, using the ORF finder algorithm (http://www.ncbi.nlm.nih.gov/projects/gorf/). Additionally, the predicted amino acid sequence was generated. To identify conserved regions and assess the degree of protein conservation among the different isolates, multiple alignments were performed utilizing the Clustal Omega program (www.ebi.ac.uk/Tools/msa/clustalo/) and the Basic Local Alimentation Search Tool (https://blast.ncbi.nlm.nih.gov/Blast.cgi). For the determination of identity and similarity between the distinct sequences, the Sequence Identities and Similarities (SIAS) program was employed (http://imed.med.ucm.es/Tools/sias).

#### B-cell epitopes prediction and peptides design

Prediction of conserved B-cell epitopes was performed using 4 different algorithms. The programs employed and the cut-off values were: BCEPred (http://www.imtech.res.in/raghava/bcepred/) with a score of 1, ABCpred (http://www.imtech.res.in/raghava/abcpred/) with a score of 0.8, IEDB (immune epitope database and analysis resource) (http://www.iedb.org/) with a score of 0.8 and EMBOSS antigenic (emboss.open-bio.org/wiki/Appdocs) with a score value of 1.10. Later, peptides containing the B-cell epitopes were designed. Finally, a BLAST analysis was conducted to confirm that the amino acid sequence of the peptides exhibited 100% identity only with the corresponding protein. Thus, selection criteria considered a maximum peptide size of 25 amino acids, specificity to *R. microplus* and sequence conservation across the 3 strains. The designed peptides were ordered for synthesis in the MAP-8 system to the company PEPTIDE 2.0 (Chantilly, VA, USA). In total, 4 peptides were synthesized with a lineal size of 19–22 amino acids, and were named chitinase 1, chitinase 2, chitinase 3 and chitinase 4. The detailed characteristics of the peptides are shown in Table 1. The MAP-8 system, in which 8 linear peptides are conjugated through specific amino acid cores, was chosen given some immunological considerations, like the increased molecular weight and the flexibility provided by the lysine core, which results in higher immunogenicity (Tam, 1988). The lyophilized peptides were solubilized in PBS buffer at a pH of 7.4, achieving a concentration of 2 mg mL^−1^ and stored at −20°C until use.

**Table 1.** Peptides sequences

Peptide	Sequence	Amino acid	Position	Antigenicity value	Allergenicity value
Chitinase 1	CYYSAWANSRPH PANYGIKDV	21	61–81	0.20 (non-predicted)	0.14 (predicted)
Chitinase 2	SADRRALFIESVLL WMKEYNLD	22	153–173	0.19 (non-predicted)	0.70 (non-predicted)
Chitinase 3	AYDLRGTWNGVT DVHPLFP	19	246–266	0.79 (predicted)	0.52 (non-predicted)
Chitinase 4	LKGNWKREFDDEG KCPYVYYR	21	348–368	0.73 (predicted)	0.31 (predicted)

Antigenicity were values obtained from the VaxiJen v2.0 server (https://www.ddg-pharmfac.net/vaxijen/) with values above the 0.4 threshold considered as probable antigens.

Allergenicity values were obtained from the AlgPred 2.0 server (https://webs.iiitd.edu.in/raghava/algpred/) with values under the 0.4 threshold considered as probable allergens.

### Animals

For the immunization experiment, 10 crossbred European cattle (*Bos taurus*) were tested. These animals were located in the experimental field ‘Valle del Guadiana’, a designated tick-free area, belonging to the National Institute for Forestry, Agricultural and Livestock Research, situated in Durango, Mexico (23°59′N, 104°37′W). The selected animals were healthy, with an initial average weight of 250 kg and managed within an extensive grazing system. For the vaccine efficacy assay, 4 cattle (*B. taurus*) from a tick-free area were purchased and confined to cattle pens located at the facilities of the Autonomous University of Queretaro (21°40′N, 100°36′W).

### Immunogenicity experiment

The PBS-diluted peptides were mixed and emulsified with oil adjuvant to prepare the vaccine doses. For each analysed peptide, 2 cattle were immunized with 100 *μ*g of the respective antigen, using a similar dose to the reported in other cattle immunization experiment using synthetic peptides (Hidalgo-Ruiz *et al*., 2022). Each vaccine dose comprised a final volume of 1 mL, evenly divided with 50% consisting of an aqueous component of PBS buffer at a pH of 7.4, wherein the antigen was diluted. The remaining half was constituted by the oil adjuvant Montanide™ ISA 201 VG (Seppic, France), emulsified by sonication. In parallel, 2 animals received control doses, consisted of equal proportions of the PBS buffer and the adjuvant. The animals were subcutaneously immunized 4 times at days 0, 21, 42 and 63. Five serum samples were collected before each injection and 14 days after the last dose.

### Serological analyses

#### Indirect ELISA

The serum samples were subjected to analysis by indirect ELISA to determine the antibody response against each peptide. Ninety-six-well ELISA plates were coated with 100 *μ*L of each peptide diluted in carbonate/bicarbonate buffer at a pH of 9.6 to a concentration ranging from 4 to 8 *μ*g mL^−1^ (previously standardized for each peptide) and incubated at 4°C overnight. After incubation, the plate wells were washed 3 times with 200 *μ*L of PBS Tween 0.05% buffer at a pH of 7.4, blocked with 200 *μ*L of 5% skim milk in PBS Tween 0.05% and incubated at 37°C and 200 rpm for 1 h. Following another round of washing, serum samples were diluted to a concentration of 1:500 in PBS Tween 0.05% and 100 *μ*L of each diluted sample was added into triplicate wells and incubated at 37°C for 1 h. The plates were washed again and then 100 *μ*L of goat anti-bovine IgG-horseradish peroxidase (Jackson ImmunoResearch, West Grove, PA, USA) was added into each well at a dilution of 1:2000 in PBS Tween 0.05%. The plates were incubated at 37°C for 1 h and a final washing step was made. Finally, 100 *μ*L of substrate (o-phenylenediamine dihydrochloride) in citrate buffer (0.1 M citric acid, 0.2 M dibasic sodium phosphate and 4 *μ*L of 30% hydrogen peroxide) was added into each well. After an incubation of 25 min at room temperature, the ELISA plates were read on an iMark Microplate Absorbance Reader (Bio-Rad, Hercules, CA, USA) at a wavelength of 450 nm. The O.D. values for the antibody results were statistically analysed with 1-way analysis of variance (ANOVA) test (*P* = 0.01) with the IBM SPSS 21 software.

#### Western blot

Total proteins extracted from 14-day-old *R. microplus* nymphs were separated by electrophoresis in an 8% SDS polyacrylamide gel (Life Science, Hercules, CA, USA). Following electrophoresis, the proteins were transferred onto a nitrocellulose membrane. The transferred membrane was treated with a blocking solution consisting of TBS-Tween 0.1% at a pH of 7.6 with 5% skim milk and incubated overnight at 4°C, under constant agitation. Next, the membrane was washed 4 times with TBS-Tween 0.1%. The serum collected from chitinase peptide 3-vaccinated cattle served as the primary antibody in a 1:500 dilution in TBS-Tween 0.1%. The other peptides were non-reactive, so they were not included in the next evaluations. The membrane was incubated for 1 h at room temperature, under constant agitation, and then washed 5 times with 1% skim milk in TBS-Tween 0.1%. Subsequently, the membrane was incubated with goat anti-bovine IgG-horseradish peroxidase (Jackson ImmunoResearch) diluted 1:10000 in the TBS-Tween-skim milk solution. The membrane underwent a further 1 h incubation at room temperature under constant stirring. After a final washing step, the membranes were revealed by chemiluminescence with ECL Western Blotting System (Bio-Rad) and visualized in the Chemi Doc MP Imaging System (Bio-Rad).

### Vaccine efficacy experiment

#### Immunization

The peptide chitinase 3 was selected for the vaccine efficacy assay. Two cattle from a tick-free area were subjected to the same vaccine dosing regimen as previously described. Similarly, 2 animals were tested as negative controls. Inoculations were carried out on days 0, 21 and 35 of the experiment, continuing until positive serum antibody levels were detected.

#### Tick infestation

Ten days after the final immunization, all 4 animals were infested each with 0.5 g of *R. microplus* larvae Media Joya strain. The ticks were applied in 2 separate chambers (0.25 g per chamber) in 2 consecutive days.

#### Data collection and analysis

Engorged female ticks were collected daily at 19–26 days post-infestation. The ticks were washed and disinfected, counted, weighted, and placed in Petri dishes for incubation at 28°C and 85% of humidity to induce oviposition. The egg masses were collected and weighted daily for a span of 10 days from the onset of oviposition. The fertility index was determined by separating 1 g of egg masses (0.5 g for bovine ticks) and allowing them to hatch into larvae. Fifteen days after hatching, the unhatched eggs and both dead and alive larvae were counted in triplicate. The tick weight was statistically analysed using the Kruskal–Wallis test (*P* = 0.05). For fertility and oviposition differences, a *t*-test was performed (*P* = 0.05). Vaccine efficacy (E) was calculated following a previously reported formula shown in Table 2 (Cunha *et al*., 2013; Ortega-Sánchez *et al*., 2020).

**Table 2.** Vaccine efficacy formula and terminology employed for tick challenge experiment

DT = % reduction in tick infestation, DW = % reduction in tick weight, DO = % reduction in oviposition, DF = % reduction in egg fertility, E = efficacy of the vaccine
The above parameters were calculated as follows:
DT = 100 [1–(NTV/NTC)], where NTV = number of adult female ticks in the vaccinated cattle and NTC = number of adult female ticks in the control cattle
DW = 100 [1–WTV/WCT], where WTV = weight average of adult females in the vaccinated cattle and WTC = weight average of adult females in the control cattle
DO = 100 [1–PATV/PATC], where PATV = weight average of the eggs per surviving tick in the vaccinated cattle and PATC = weight average of the eggs per surviving tick in the control cattle
DF = 100 [(HC–HT)/HC], where HC = viability index in control ticks and HT = viability index in challenged ticks
Vaccine efficacy (E) was calculated as 100 [1–(CRT × CRO × CRF)], where CRT = NTV/NTC = reduction in the number of adult female ticks compared to the control ticks, CRO = PATV/PATC = reduction in the egg laying capacity of the survived ticks compared to the control ticks and CRF = PPLOV/PPLOC where PPLOV is the mean of larval weight per gram of eggs in the challenged ticks and PPLOC is the mean of larval weight per gram of eggs in the control ticks

## Results

### Bioinformatics

**Figure 1.**
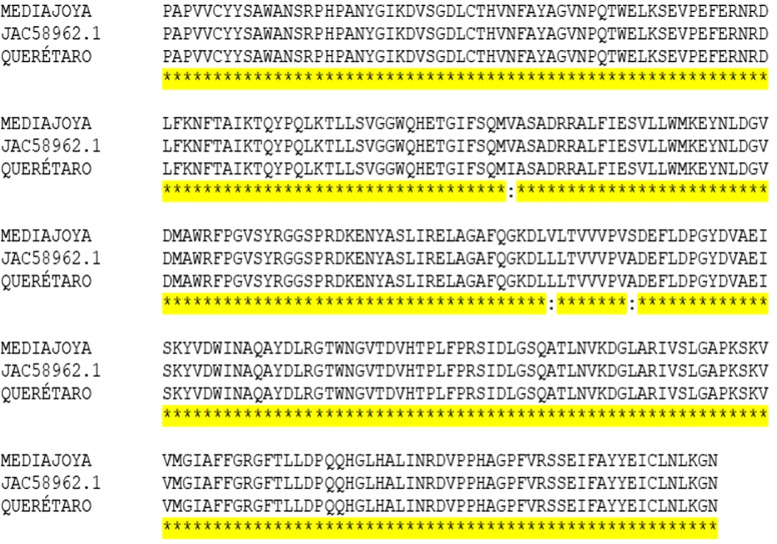
Multiple alignment of the *chitinase* gene for the Media Joya, Queretaro and the reference strain JAC58962.1. The conserved regions of the different sequences are marked in yellow. The CLUSTAL O (1.2.4) multiple sequence alignment program was used.

**Figure 2.**
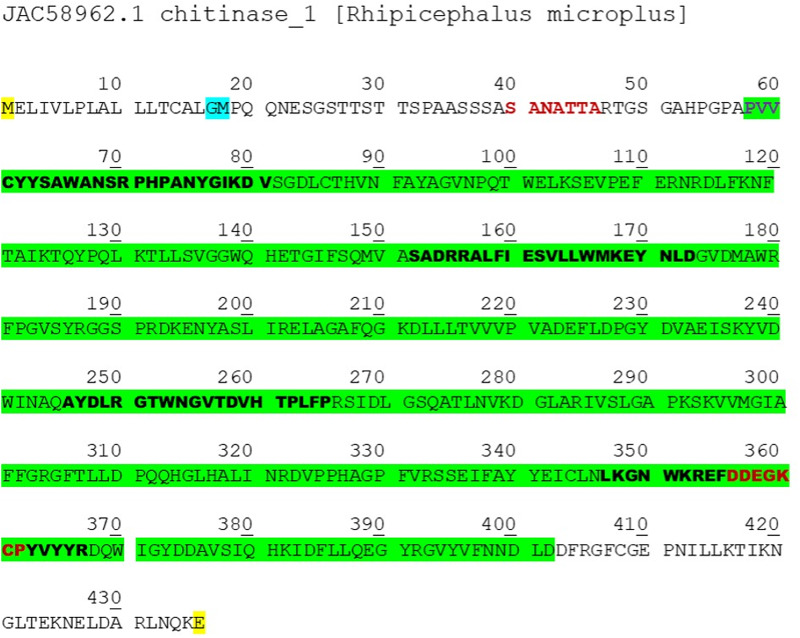
Sequence of amino acids for the chitinase sequence. Yellow indicates the location of the start and stop codon; blue marks the end of the signal peptide; red indicates the location of the sense and antisense primers (on the DNA coding sequence); the Glyco 18 domain is shown in green; the selected peptides are highlighted in black. There is no transmembranal helix.

The 3 evaluated sequences corresponded to Queretaro strain, the Media Joya strain and the reference strain JAC58962.1, obtained from the *R. microplus* transcriptome. These chitinase sequences from the 3 strains were subjected to alignment (Fig. 1). The predicted chitinase coding sequence comprised 1311 bp, out of which 975 bp were successfully amplified and 890 bp were sequenced (Fig. 2). Later, an average identity percentage of 99.32% and a similarity of 99.84% were noted (Table 3).

**Table 3.** Similarity percentages between the amino acid sequences of the different sequences for chitinase

Isolates	JAC58962.1	Media Joya	Queretaro
JAC58962	100%		
Media Joya	99.32%	100%	
Queretaro	99.66%	98.98%	100%

The SIAS program, sequences identities and similarities, was used.

Four peptides, situated within conserved regions of the protein, were identified using 4 B-cell epitope predictor tools. These peptides were constructed to have lengths ranging from 19 to 22 amino acids and were positioned at different segments of the protein sequence. The sequences of the peptides, their length, position, antigenicity and allergenicity values are shown in Table 1.

### Determination of immunogenicity of peptides

**Figure 3.**
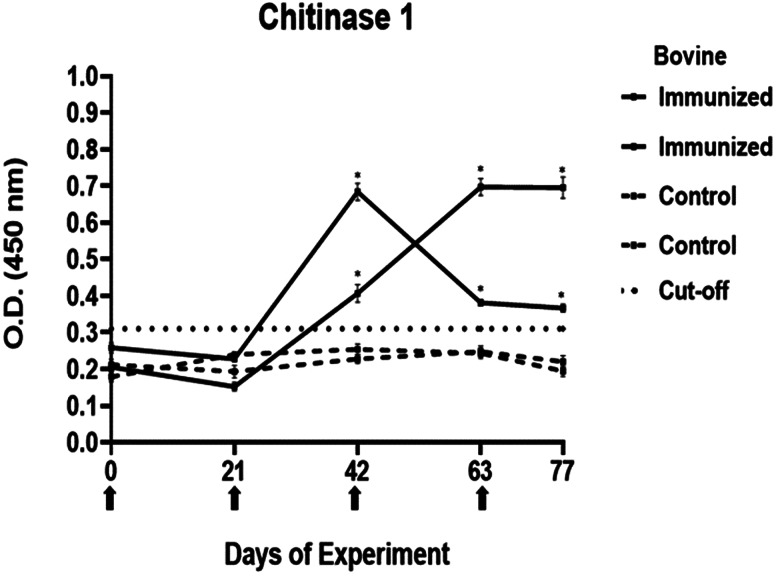
Antibody response in vaccinated cattle. Bovine serum antibody production to chitinase 1 was determined by indirect ELISA. 1:500 serum dilution was analysed at O.D. 450 nm. One-way ANOVA was used to compare the readings between the 4 cattle (*P* = 0.01). Days of immunizations are indicated with arrows.

**Figure 4.**
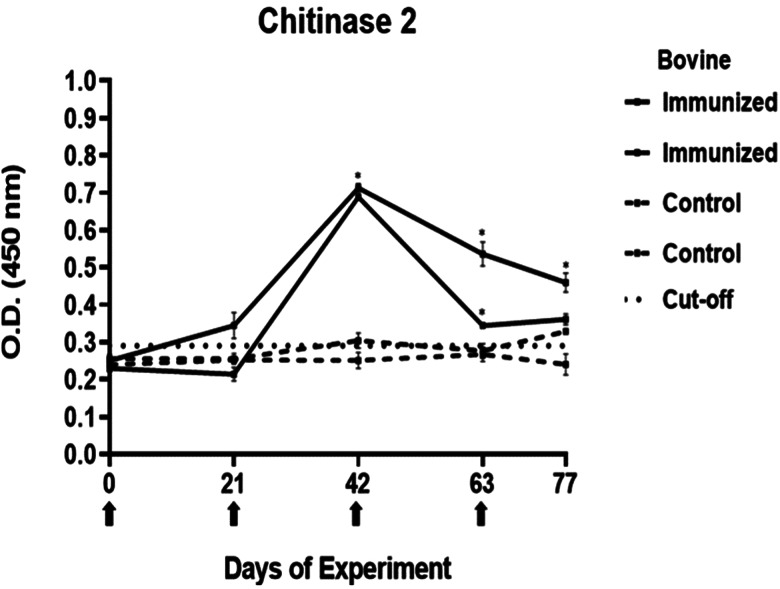
Antibody response in vaccinated cattle. Bovine serum antibody production to chitinase 2 was determined by indirect ELISA. 1:500 serum dilution was analysed at O.D. 450 nm. One-way ANOVA was used to compare the readings between the 4 cattle (*P* = 0.01). Days of immunizations are indicated with arrows.

**Figure 5.**
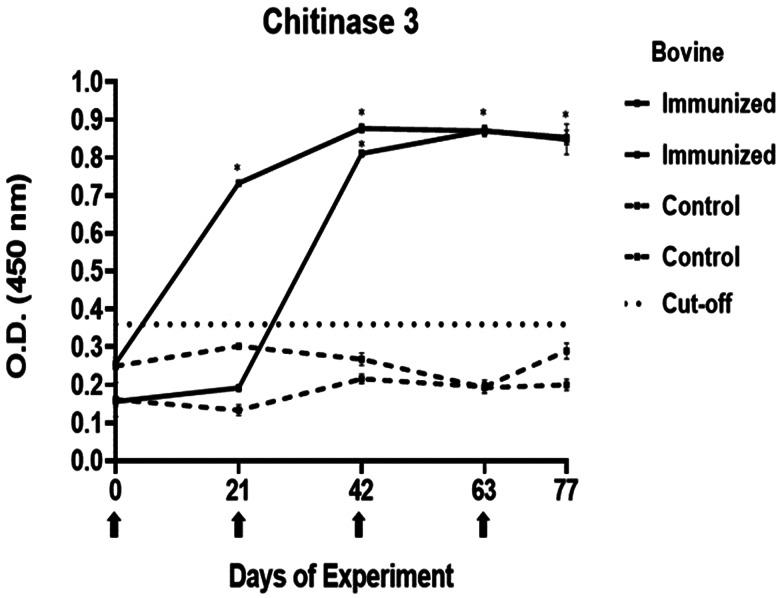
Antibody response in vaccinated cattle. Bovine serum antibody production to chitinase 3 was determined by indirect ELISA. 1:500 serum dilution was analysed at O.D. 450 nm. One-way ANOVA was used to compare the readings between the 4 cattle (*P* = 0.01). Days of immunizations are indicated with arrows.

**Figure 6.**
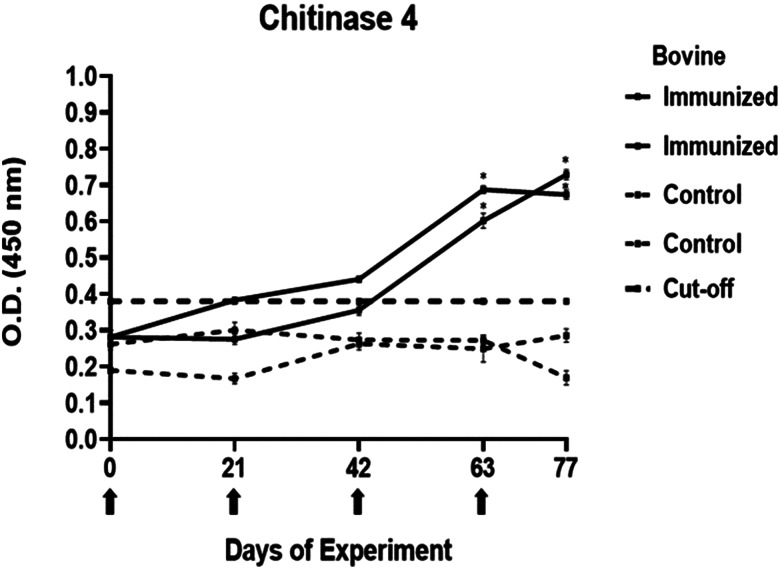
Antibody response in vaccinated cattle. Bovine serum antibody production to chitinase 4 was determined by indirect ELISA. 1:500 serum dilution was analysed at O.D. 450 nm. One-way ANOVA was used to compare the readings between the 4 cattle (*P* = 0.01). Days of immunizations are indicated with arrows.

**Figure 7.**
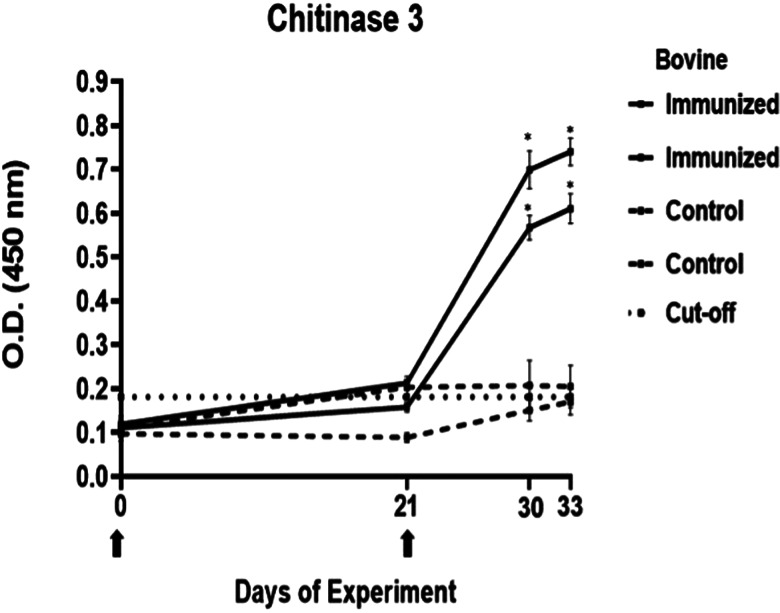
Antibody response in vaccinated cattle before larvae infestation. Bovine serum antibody production to chitinase 3 was determined by indirect ELISA. 1:500 serum dilution was analysed at O.D. 450 nm. One-way ANOVA was used to compare the readings between the 4 cattle (*P* = 0.01). Days of immunizations are indicated with arrows.

The antibody response in immunized cattle was evaluated through the standardization of indirect ELISA tests for each peptide. The antibody level results are depicted in Figs 3–7. Notably, the chitinase 3 peptide exhibited immunogenic potential by achieving the following criteria: (1) both immunized animals were positive above the cut-off with a maximum of 2 doses, (2) the antibody response increased or stood above the cut-off after each subsequent immunization, (3) the immune sera were statistically different from the control sera and (4) the control sera remained below the cut-off threshold.

Conversely, the antibody response observed with peptides 1, 2 and 4 showed a different pattern. The main and common characteristic that led to a peptide discard was the decline in antibody levels below the cut-off after the third immunization. In the case of chitinase 4, the reason for excluding the peptide stemmed from the number of inoculations required before achieving a positive result.

### Western blot

**Figure 8.**
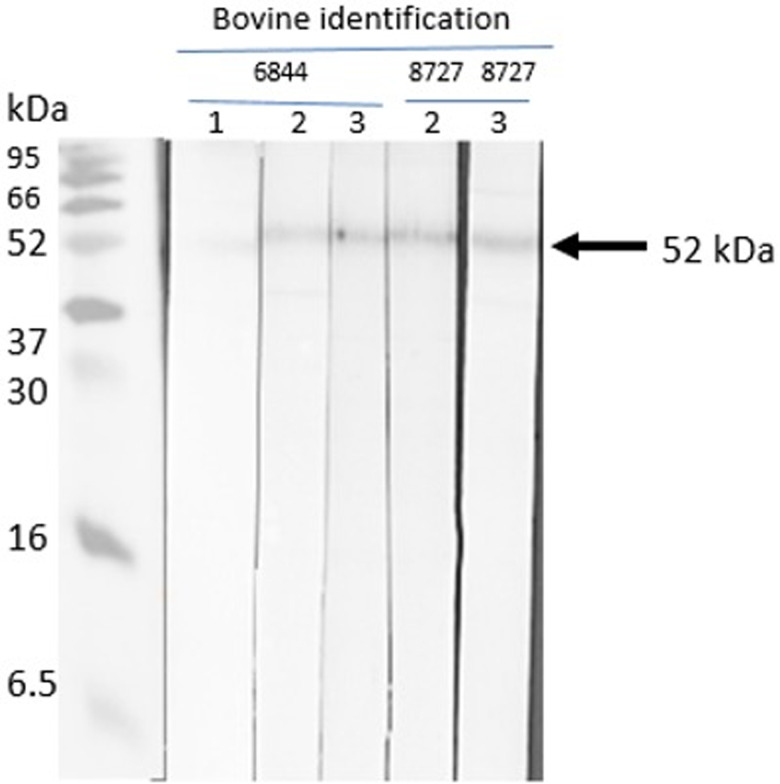
Western blot with chitinase 3. Sera from chitinase 3 vaccinated cattle (cattle 6844 and 8727). Lane 1: pre-immune bovine serum; lane 2: third inoculation, post-immune bovine sera; lane 3: fourth inoculation, post-immune sera.

**Figure 9.**
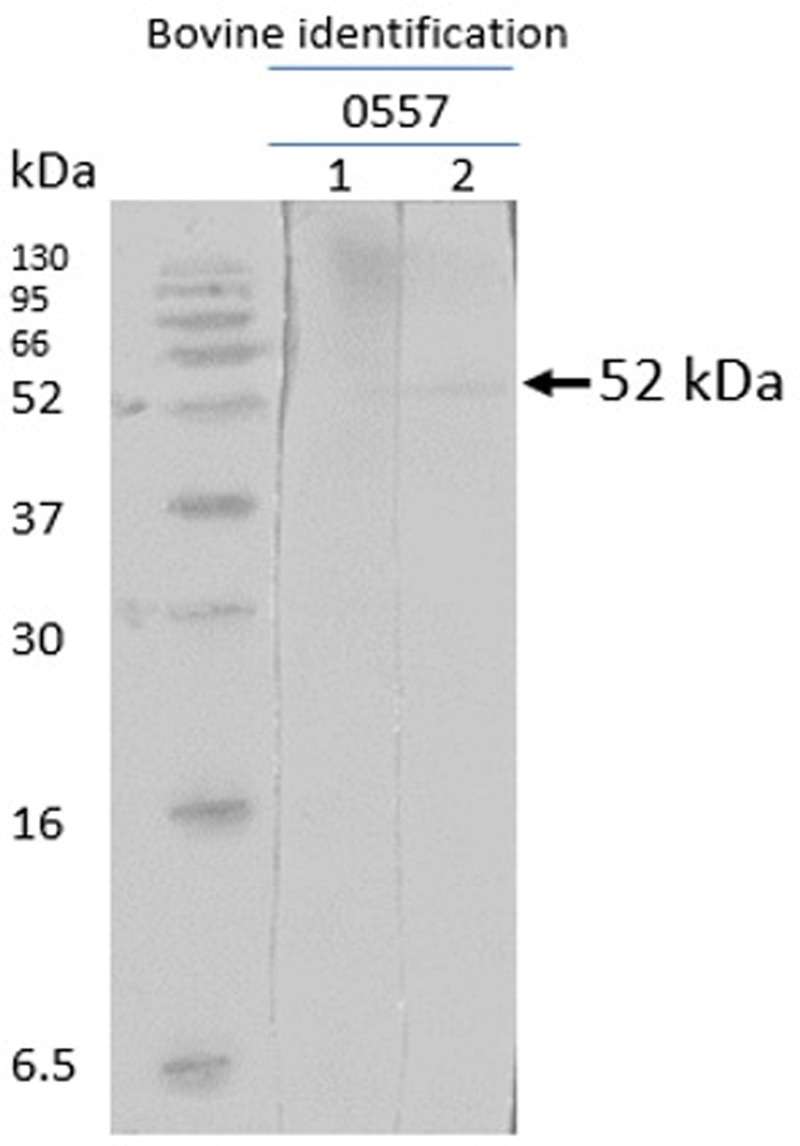
Western blot with chitinase 3. Sera from chitinase 3 vaccinated cattle after infestation (cattle 0557) lane 1, pre-immune and pre-infestation bovine serum; lane 2, post-immune and post-infestation bovine serum.

The native *R. microplus* chitinase, with a predicted molecular mass of 52 kDa, was recognized by sera of cattle immunized with chitinase 3, thus indicating that the peptide-induced antibodies recognize the full protein obtained in nymph extracts. The pre-immune sera of the cattle did not detect the native chitinase (Figs 8 and 9). The other 3 peptides were non-reactive.

### Experimental challenge

#### Effect of immunization on tick survival and weight

**Figure 10.**
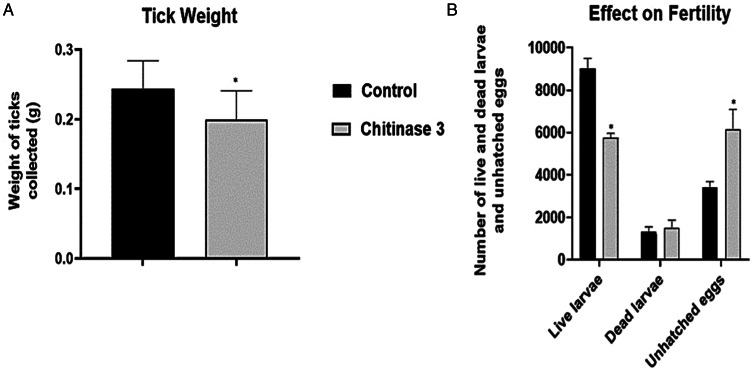
Effect of chitinase 3 treatment on biological parameters. (A) Effect of chitinase 3 on tick weight. (B) Effect of chitinase 3 on fertility. In (A) and (B), black bars correspond to control animals and grey bars correspond to treated animals. The results were compared to a *t*-test with a significance value of 95%.

All animals used in the tick infestation experiment were immunized 3 times with the correspondent doses until detecting positive antibody levels. Chitinase 3 reduced the number of ticks (*n* = 1598) in 19% compared to the control (*n* = 1967). On tick weight, a 17% decrease was observed in ticks fed on chitinase peptide 3-vaccinated cattle (0.207 ± 0.035 g) compared to the control (0.249 ± 0.038 g). The effect of immunization with peptide 3 on tick weight is shown in Fig. 10.

#### Effect of immunization on oviposition and fertility

The average oviposition mass per tick challenged (0.09 ± 0.03 g) was lower than in control ticks (0.13 ± 0.03 g), so was the total oviposition mass (229.31 g in control compared to 125.09 g in challenged ticks). For fertility, the ticks that fed with the immune serum had a lower fertility rate, increasing the numbers of dead larvae and unhatched eggs. The effect of immunization with chitinase 3 on tick fertility is shown in Fig. 10.

#### Chitinase 3 vaccine efficacy

The result of the efficacy formula using the chitinase 3 peptide was 71.18% and the data are shown in Table 4 .

**Table 4.** Data of experimental infestation challenge and protective efficacy of chitinase 3 peptide against *Rhipicephalus microplus*

Cattle	Engorged females (*n*)	Average weight (g)	Surviving oviposition ticks (*n*)	Total oviposition mass (g)	Fertility index
Control 1	1023	0.253 ± 0.042	1654	229.31	0.656
Control 2	944	0.244 ± 0.022			
Vaccinated 1	834	0.209 ± 0.038	1371	125.09	0.426
Vaccinated 2	764	0.203 ± 0.034			
**Efficacy**					
	**DT**	**DW**	**DO**	**DF**	**E**
Chitinase 3	18.76%	16.87%	33.92%	35.06%	71.18%

**Figure 11.**
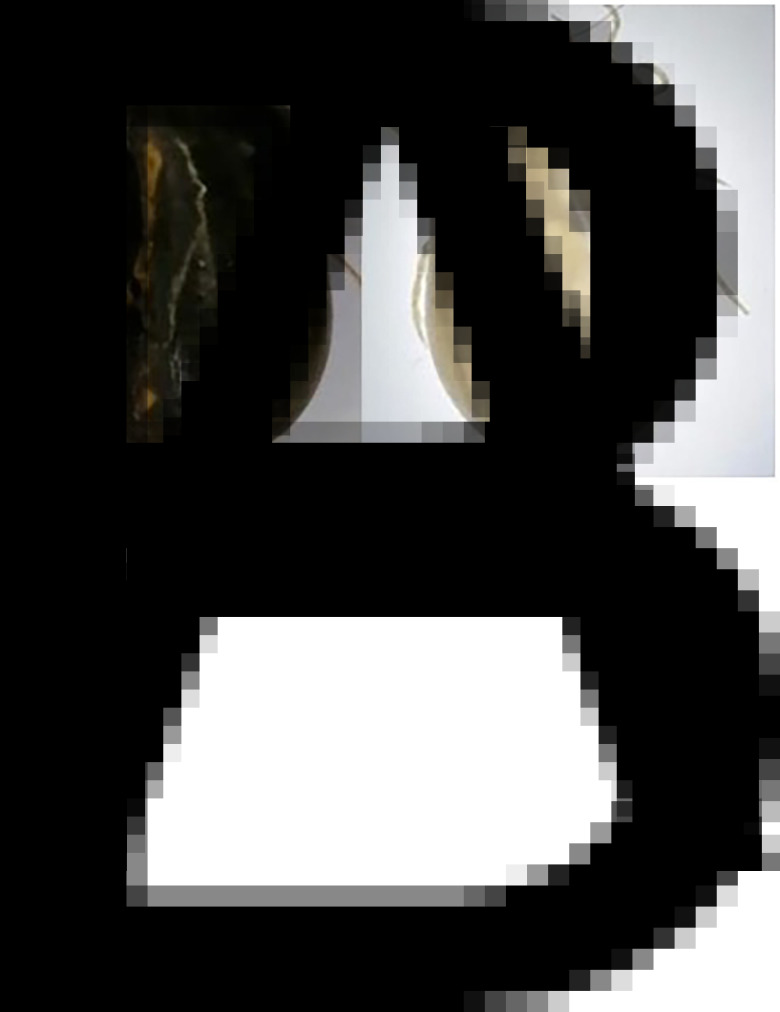
Macroscopical effect of chitinase 3 on engorged ticks. (A) Control engorged ticks. (B) chitinase 3 treated engorged ticks.

## Discussion

*Rhipicephalus microplus* control presents one of the most significant challenges to the tropical and subtropical livestock industry, contributing to reduced meat and milk production, the transmission of tick-borne diseases and resulting in yearly economic losses of billions of dollars (Jongejan and Uilenberg, 2004; Jonsson, 2006; Singh *et al*., 2022). The identification of conserved immunological determinants could lead to the development of a new alternative of tick vaccine. It has been established that a potential tick antigen must be tested to evaluate its viability as a vaccine option, by meeting criteria as immunogenicity, impact on tick biology and vaccine efficacy (Elvin and Kemp, 1994; Willadsen, 2004; de la Fuente and Contreras, 2015; Kasaija *et al*., 2023).

This work marks the first research regarding the allelic variability of the *chitinase* gene across distinct isolates of *R. microplus*. This gene encodes an arthropod-specific protein previously pointed as a potential candidate for tick vaccines (You and Fujisaki, 2009). The significance of choosing a conserved antigen arises from the observed variability in the Bm86 sequence, which might contribute to the variable efficacy seen in recombinant vaccines against *R. microplus* (García-García *et al*., 1999; Kaewmongkol *et al*., 2015). Furthermore, Freeman *et al*. (2010) suggest an inverse correlation between vaccine efficacy and locus variation. To address this concern, the amplification and sequencing of *chitinase* were carried out, and the results were compared with reference strains.

According to the data published by García-García *et al*. (1999), variability greater than 3.4% between different sequences of the same protein is enough to elicit an inefficient immune response. The demonstrated 0.16% of similarity and 0.68% of identity for chitinase highlight the low variability between the amino acid sequences of the 3 isolates. Hence, chitinase can be considered as a conserved antigen in *R. microplus* populations, thereby fulfilling one of the desirable criteria. More sequences from different strains are needed to confirm such conservation.

The use of synthetic peptides is not a new strategy in the development and evaluation of tick vaccines (Patarroyo *et al*., 2002). For its part, the use of MAPs technology could be novel. There are some works that have used peptides in MAPs for the evaluation of vaccine antigens against bovine babesiosis (Hidalgo-Ruiz *et al*., 2022).

We used Montanide ISA 201 VG as the primary adjuvant for immunization of antigen to cattle because of the existing evidence demonstrating that it aids to elicit elevated humoral responses in cattle, and for being an inducer of IgG2 responses, which is the antibody pointed as crucial for the anti-tick effect. Furthermore, some articles have described that Montanide ISA 201 VG provides a faster action in the development of antibody responses, and the duration of the humoral response is similar when compared to other Montanide like the ISA 50, 61 and 206 (Dar *et al*., 2013; Ibrahim *et al*., 2015; Osman *et al*., 2020).

The immunogenicity tests yielded a single peptide selected for the subsequent experiment. The immunizations with MAP-8 peptides resulted in high antibody levels after 2 doses for peptides 1–3, whereas only with peptide 3, the antibody response increased or stood the same after the next doses. In the case of peptide 4, 4 immunizations were required to detect a statistically significant difference between the immune and control sera. Failures in immune response might be caused by different reasons, including operational factors, animal-related factors linked to immunological traits and health status (Roth, 1999; Maritz-Oliver *et al*., 2012). Additionally, the decline in antibody response has been associated with disruptions in vaccine stability or even self-tolerance to the antigen (Almazan *et al*., 2010). Peptide 3 was selected for its promising immunogenic outcomes. The literature suggests that a substantial and increasing immune response to a tick antigen could be positively correlated to the antibody levels that it elicits (de la Fuente *et al*., 1995; de la Fuente *et al*., 1998; Merino *et al*., 2011). In the case of chitinase, a high antibody response is needed for adequate efficacy.

For certain antigens, like chitinase enzymes, their time of expression is limited time during the tick life, so an elevated response and lots of circulating antibodies are crucial (Maritz-Oliver *et al*., 2012). A possible explanation for just 1 peptide being truly immunogenic could also be set in the deviation rate of the bioinformatic tools.

Chitinases are enzymes with a wide range of functions, including moulting success, exoskeleton separation and even digestion in some animal species (Merzendorfer and Zimoch, 2003; Arakane and Muthukrishnan, 2010). In *H. longicornis* ticks, vaccination with recombinant chitinase resulted in a 20% decrease in nymphal moulting success and tick mortality during this stage. In the current study, a mortality rate of 18.75% was observed, but the specific tick stage affected could not be discerned (You and Fujisaki, 2009). It could be inferred that the partial mortality might stem from the impairment of a single enzyme involved in apolysis and ecdysis processes, but other hydrolytic enzymes may take a role in these stages. Another observed effect was the decline in tick weight, perhaps due to some blocking action in the growth and development of cuticle and guts.

You and Fujisaki (2009) previously reported the chitinase vaccination on tick's oviposition, but the percentage of damage now found is higher than the one they described. The number of eggs laid by engorged ticks is linked to their weight. The increase in dead larvae and unhatched eggs could be attributed to a hindrance in the chitinolytic action during the hatching of new larvae (Arnold *et al*., 1993; Moreira *et al*., 2007; Farnesi *et al*., 2015).

Moreover, certain physical alterations were noticed in a fraction of the collected ticks, with notorious changes in the cuticle. The cuticle of these ticks exhibited translucency and friability, rendering the tick less resistant and increasing the proportion of engorged ticks that perished with minimal or no oviposition. This effect might stem from the fact that certain chitinases not only possess hydrolytic capabilities but also exert a regulatory influence on chitin metabolism (Arakane and Muthukrishnan, 2010; Zhu *et al*., 2016).Examples of these affected ticks are depicted in Fig. 11.

Although these results are promising, further studies including a proper vaccine efficacy trial would be needed for a better understanding of the potential of this antigen alone as a vaccine for *R. microplus* ticks. However, in future experiments, chitinase 3 could be included of a multiantigenic tick vaccine and tested in a larger group of cattle.

## Conclusion

In the present study, the molecular conservation of chitinase was demonstrated and encouraged the design of peptides with B-cell epitopes. Additionally, the testing of a peptide with B-cell epitopes indicates the possible role of this antigen as a vaccine candidate. The chitinase 3 peptide exhibited promising immunogenicity results and affected biological parameters of ticks, mainly on oviposition and fertility, for an overall vaccine efficacy of 71.18%. This study is the first to report the vaccine potential of *R. microplus* chitinase and the first step for the integration of a multiantigenic novel vaccine.

## Data Availability

The data will be available under request.
